# RETHINKING RURAL GERONTOLOGY THROUGH A DEWEYAN PRAGMATIST PERSPECTIVE

**DOI:** 10.1093/geroni/igz038.1476

**Published:** 2019-11-08

**Authors:** Malcolm Cutchin, Malcolm P Cutchin, Graham D Rowles

**Affiliations:** 1 Wayne State University, Detroit, Michigan, United States; 2 University of Kentucky, Lexington, Kentucky, United States

## Abstract

Rural aging as we have conceived of it in the gerontological literature of the past 50 years no longer exists, if it ever did. In this presentation, we contribute toward a reframing of the discourse on rural aging through a critique of established views of rural aging as an ecological, cultural, and phenomenological experience. We argue that each view is limited in its ability to encapsulate the essence of rural living and community. Our critique provides a context for a dynamic perspective on rural aging that embraces the situational uniqueness of each rural environment. We introduce that perspective, based in John Dewey’s philosophy, and grounded in the idea of situationally defined manifestations of place integration within an ever-changing milieu. We conclude with a discussion of key implications, including how this perspective reshapes the roles of researchers and older rural residents in the process of ongoing rural gerontological inquiry.

